# Comparison of the accuracy of conventional and digital radiography in root canal working length determination: An invitro study

**DOI:** 10.15171/joddd.2017.029

**Published:** 2017-09-20

**Authors:** Mohammad Mahdi Yaghooti Khorasani, Hamed Ebrahimnejad

**Affiliations:** ^1^Department of Endodontics, School of Dentistry, Rafsanjan University of Medical Sciences, Rafsanjan, Iran; ^2^Department of Oral and Maxillofacial Radiology, School of Dentistry, Kerman University of Medical Sciences, Kerman, Iran

**Keywords:** Digital radiography, endodontics, root canal therapy

## Abstract

***Background.*** Digital radiography has widespread use in endodontics. Determining a correct working length is vital for a proper endodontic therapy. The aim of this study was to compare the accuracy of conventional and digital radiographic techniques for root canal working length determination.

***
Methods.*** After determining the real working lengths of 50 permanent maxillary central incisors (gold standard), the conventional (E- and F-speed films) and digital (CCD, PSP) images were obtained using the parallel technique. The mean registered working length of each modality was compared with the other and with the gold standard using one-way ANOVA at P<0.05.

***
Results.*** No significant difference was found between the recorded working length values using the conventional and digital radiographic techniques (P=0.828).

***
Conclusion.*** Within the limitations of this study, it was concluded that there was no difference between the measurement accuracy of CCD, PSP and conventional imaging techniques in root canal working length determination.

## Introduction

Dental radiography plays a fundamental role in endodontic treatment. A comprehensive evaluation of root canal therapies cannot be accomplished without the help of imaging methods.^1^ Conventional periapical (PA) radiographs have always been the most common modality for this purpose.^2^ In addition, digital PA radiography is a newer technique with several advantages over the conventional one. Lower radiation dose, less time-consuming nature, the ability to process, modify, save and transfer the images and elimination of developing procedures are among these advantages, which have made the digital modality a favorable choice for endodontic therapy. However, the conventional technique has better spatial resolution and is less expensive.^3-6^

Precise root canal working length (WL) determination is an essential prerequisite for a successful root canal therapy (RCT). Endodontic mishaps such as apical perforation, improper cleaning and shaping or over-/under-filling may occur following an inaccurate WL determination, which can lead to RCT failure.^7,8^

There are different methods available for estimating the length of root canal such as tactile sensation, assessment of preoperative radiographs and electronic apex finders. The PA radiography, if used properly, can be a reliable and exquisite method for WL determination.,^9^ A parallel PA radiograph with an optimum contrast can readily illustrate the WL.^10^ The most common radiographs are conventional E- and F-speed films, alongside digital CCD (Charge Coupled Device) and PSP (Photo-Stimulable Phosphor Plates) images. Since in direct digital radiographic techniques such as CCD, the image is immediately observable after exposure, CCD seems to be more appropriate than PSP for endodontic therapies. However, the thickness and rigidity of CCD sensors limit their utility, whereas PSPs are more convenient and acceptable by patients.^6,11,12^ Therefore, choosing the best method could be controversial.

The diagnostic accuracy of conventional and digital PA radiographs has widely been investigated. Mostly, digital methods are authentic modalities with no significant difference from conventional ones.^7,13,14^

With the vast increase in the use of conventional and digital PA radiographs in endodontic treatments, we decided to compare the accuracy of the two approaches in root canal WL determination in an in vitro research.

## Methods

In this in vitro study, 50 permanent and mature single-rooted maxillary central incisors were selected and investigated clinically and radiographically to be free of any fracture, internal/external resorption, extreme calcification, dilaceration or significant root curvature. The apical foramina of all these single-canal teeth were almost coincident with their anatomic apices.

After cleaning and disinfecting (5.25% sodium hypochlorite, Kimidaru, Iran), the access cavity was prepared (round and fissure burs, D&Z, Sydney, Australia) and the teeth were numbered consecutively. In order to obtain the actual WL (gold standard) a #15 K-file (Mani, Nakaakutsu, Japan) was placed tip-to-tip with the apical foramen. This was confirmed by a magnifying loupe. The rubber stop was carefully positioned to the reference point (incisal edge) and the file was removed from the root canal. The distance between the stopper and the file tip was measured with a caliper with an accuracy of 0.1mm (Mitutoyo, Tokyo, Japan) by an endodontist and a maxillofacial radiologist and then 1 mm was subtracted from that point to establish the actual WL (Gold_1_). The file was then placed back in the canal and fixed in place with Cavit dressing (Altstätten, Switzerland). A week later, this procedure was repeated on all the samples randomly (Gold_2_).^2,4,15^

**Figure 1:**
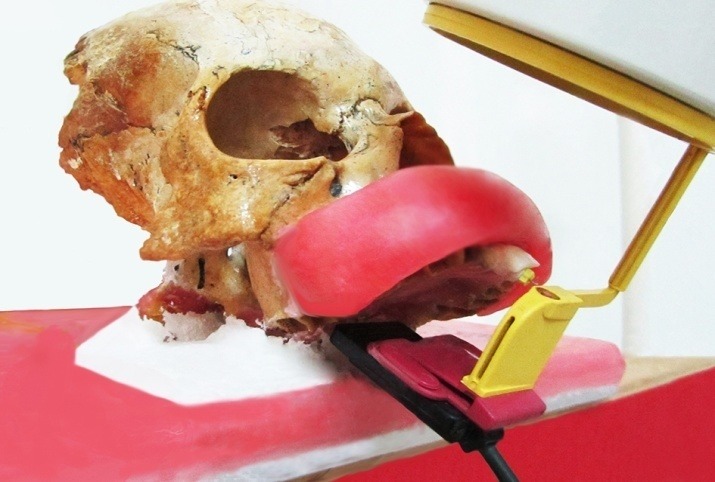
Image capturing from the embedded tooth in the mounted skull.

Each tooth was mounted securely in the maxillary central incisor socket of a dry skull, which was fixed on a flat panel by plaster. This panel had a specified constant position and the radiography tube head was immobile during the entire imaging process. Each tooth was embedded in bone to the depth of the cementoenamel junction. In order to simulate soft tissue, the labial and palatal aspects of the maxilla were covered with eight layers and one layer of base plate wax at a consistent thickness, respectively.^16-18^ A Rinn film holder (Kerr, Germany) was fixed to the x-ray tube cone at a 5-cm distance from the embedded teeth (Figure 1).

The conventional radiographs were obtained with #2 E- and F-Speed intraoral films (Eastman-Kodak Co. Rochester, NY, USA) using a Prostyle dental x-ray machine (Planmeca, Helsinki, Finland) operated at 63 kVp, 8 mA and 0.25 s. All the films were developed with an automatic film processor (Hope Dental Max, Hope X-ray Products, USA) at the same time and temperature. The CCD sensor (De Gotzen, Italy) and PSP plate (Soredex, Helsinki, Finland) were exposed at 60 kVp, 7 mA and 0.06 s. The radiographs were all captured using the parallel technique.

**Figure 2:**
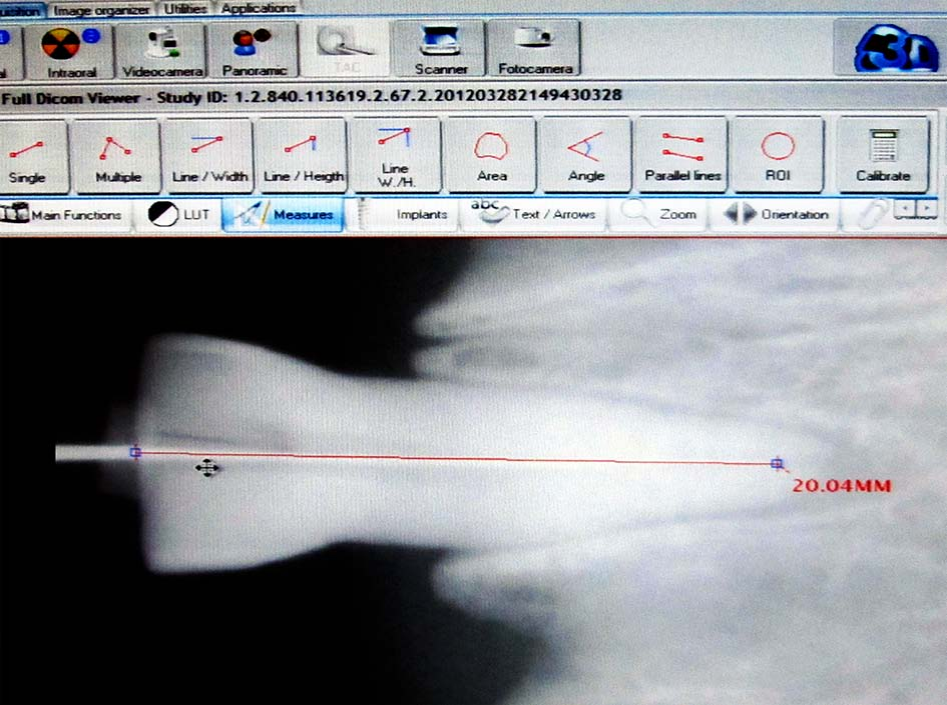
Measuring the working length in a CCD image using SOPRO software.

On the conventional radiographs (E- and F-speed films), the distance between the incisal edge and file tip was measured with a caliper by an endodontist and a maxillofacial radiologist (E_1_, F_1_). This procedure was repeated a week later (E_2_, F_2_). The same process was carried out on CCD and PSP images using SOPRO® (Sopro Imaging, Sopro SA, La Ciotat, France) and Digora® (Digora for Windows 2.0, Soredex Medical Systems, Helsinki, Finland) software programs, respectively (CCD_1_, CCD_2_; PSP_1_, PSP_2_) (Figure 2). The measurements were all made in a semi-dark room under constant observational conditions. No visual enhancement was performed on digital images. These values were further assessed by means of statistical tests.

In order to ensure the consistency of the measuring accuracy of caliper and digital software programs’ measuring tools, a conventional, CCD and PSP image were obtained from an endodontic gauge‏.The same length was measured on the three images and no difference was noticed.

### Statistical analysis

The correlation between the observers was assessed using Pearson's correlation test. Kolmogorov–Smirnov test showed the normality of data distribution. One-way ANOVA was used to compare the measuring accuracy of conventional and digital radiographs. Data were analyzed with SPSS 18 (SPSS Inc., Chicago, IL, USA) and the significance level was set to P<0.05.

## Results

There was a significant correlation between the observers in conventional and digital radiographic techniques (P<0.001). The mean data obtained at the first and second week were considered as reference values for analysis (Table 1).

**Table 1:** The mean measured working length values

**Group**	**N**	**Mean (Std. deviation)**
**Gold**_1_	50	21.57(2.32)
**Gold**_2_	50	21.47(2.22)
**E**_1_	50	21.88(2.29)
**E**_2_	50	21.87(2.25)
**F**_1_	50	22.02(2.27)
**F**_2_	50	21.69(2.27)
**CCD**_1_	50	21.59(2.22)
**CCD**_2_	50	21.56(2.17)
**PSP**_1_	50	21.69(2.23)
**PSP**_2_	50	21.59(2.17)

Gold=Gold standard; E=E-speed film; F=F-speed film

In order to compare the measuring accuracy of different radiographs, the mean WL values (Gold, E, F, CCD and PSP) were compared. No significant difference was observed between the conventional, digital and gold standard values (P=0.828).

## Discussion

Accurate root canal WL determination, a basic step in endodontic therapy, is accomplished by various techniques, using radiography, apex finders or tactile sensation.,^19,20^ In this context, digital and conventional radiographs have been compared in vitro. The results of the present study showed no significant difference between the accuracy of the two techniques and the actual measurements.

In an in vitro and in vivo study which compared digital radiography (radiovisiography) with conventional method (D-Speed film), no significant difference was observed in the estimated canal lengths using the two modalities.^21^ Shearer et al^22^ reported no significant difference between conventional films and enhanced radiovisiography in estimating the root canal length. In an in vitro comparison of premolar root lengths, CCD, PSP and E-speed films exhibited similar measurement accuracy,^23^ consistent with the results of the present study.

A majority of studies have compared the two digital and conventional imaging systems in measuring the WL of curved canals. Burger et al^24^ evaluated direct digital radiography (DDR) versus conventional radiography for determining root canal length in curved canals. Although both techniques were deficient in measuring the true root canal length, there was no significant difference between the conventional and digital radiographs. Another in vitro study on 30 curved mesiobuccal root canals in molars compared conventional and PSP images in estimating the root canal length. The measurements for determining WL of curved canals obtained on either conventional or digital images were similar.^2^ Another research on 20 mesiobuccal canals from maxillary molars with moderate-severe curvatures and 20 canals form anterior teeth yielded the same results. Five radiographs were taken for each canal. Three conventional radiographies were obtained by using different processing techniques: Manual, automatic, and monobath solution. The two other digital images were captured using CCD and PSP receptors. The results suggested that the accuracy of digital and conventional radiographic techniques was comparable.^25^ However, some authors believe that digital systems are more accurate in canals with curvatures >25 degrees.^26^ The discrepancy might be due to the different measuring methods used. Some studies have proved that conventional film is superior in WL measurement when compared to digital systems.^27-30^ This is mostly due to the old digital systems. On the other hand, some other studies have shown digital systems to be superior to conventional films.^31,32^ The numerous features available for image enhancement in digital systems might justify these findings. Computerized enhancement is more useful when detecting file tip positions, especially of small sizes. This is one reason for suggesting digital radiography in endodontic practice, albeit it mostly shows similar accuracy with the conventional one. Furthermore, digital radiography has lower radiation dose and is less time-consuming.^5^

Apex-locators are also compared with radiography in determining the root canal WL. Some researchers claim that the new generation apex finders could estimate the WL better than radiography.^33^ However, it totally depends on the accuracy of the technique used.

## Conclusion

The measurement accuracy of CCD, PSP and conventional films in WL determination exhibited no difference between these techniques. However, because of the advantages of digital systems (e.g. radiation dose reduction or image enhancement), it is preferable to the conventional one.

## Acknowledgments

The authors would like to express their gratitude to Dr. Entezari and Dr. Darabinasab for sharing their pearls of wisdom and efforts with us during the course of this research.

## Authors’ contributions

YKMM collected data and interpreted them. EH analyzed data analysis and drafted and revised the manuscript. Both authors have read and approved the final manuscript.

## Funding

Rafsanjan School of Dentistry financially supported the present study.

## Competing interests

The authors declare no competing interests with regards to the authorship and/or publication of this article.

## Ethics approval

The study was approved by Rafsanjan University of Medical Sciences.
